# Dose–Response Effect of Watermelon Consumption on Ambulatory Blood Pressure in Adults with Elevated Blood Pressure: A Randomized Controlled Pilot Trial

**DOI:** 10.3390/nu17193073

**Published:** 2025-09-26

**Authors:** Kanishka Singh, Huiling Liao, Indika Edirisinghe, Britt Burton-Freeman, Amandeep K. Sandhu

**Affiliations:** 1 Department of Food Science and Nutrition, Center for Nutrition Research, Institute for Food Safety and Health, Illinois Institute of Technology, Chicago, IL 60616, USA; 2 Department of Applied Mathematics, Illinois Institute of Technology, Chicago, IL 60616, USA; 3 Center for Food Chemistry and Packaging, Institute for Food Safety and Health, Illinois Institute of Technology, Chicago, IL 60616, USA

**Keywords:** watermelon, L-citrulline, L-arginine, blood pressure (BP), ambulatory blood pressure (AMBP) monitor

## Abstract

**Background/Objectives**: Watermelon (*Citrullus lanatus*) is a natural dietary source of L-citrulline and L-arginine, the two amino acids involved in nitric oxide (NO) production and vasodilation. Pre-clinical and clinical studies using isolated amino acids or watermelon extracts suggest blood pressure (BP)-lowering potential; however, limited research has been conducted on the impact of watermelon flesh (WM) on BP in adults at risk for hypertension. Therefore, the primary objective of this study was to assess the effect of daily WM intake for four weeks on 24 h ambulatory BP in adults with elevated blood pressure. The secondary outcomes of this study include changes in glucose and insulin markers, lipid profile, NO, L-citrulline, L-arginine, asymmetric dimethylarginine (ADMA) concentrations, and the L-arginine/ADMA ratio. **Methods**: In this randomized, placebo controlled parallel study design, 39 adults (age: 41 ± 14 years, BMI: 31 ± 6 kg/m^2^, mean ± SD) with elevated BP were randomly assigned to one of three groups for a 4-week intervention: control (0 g WM), WM-1 cup (152 g/day), or WM-2 cups (304 g/day). Ambulatory BP was measured over 24 h at baseline and the end of the intervention period. Fasting plasma samples were analyzed for metabolic biomarkers on a clinical analyzer and NO using a colorimetric assay. L-citrulline, L-arginine, and ADMA were analyzed using an ultra-high-performance liquid chromatography triple quadrupole mass spectrometer (UHPLC-QQQ-MS/MS). Statistical analyses were conducted using SPSS software (IBM SPSS Statistics, Version 29.0.0). **Results**: After 4 weeks, mean 24 h ambulatory BP was 130.2 ± 3.9 mm Hg (control), 130 ± 3.2 mm Hg (WM-1 cup), and 124.9 ± 3.9 mm Hg (WM-2 cups), with no statistically significant differences between study interventions (*p* > 0.05). Similarly, no significant changes were observed in fasting plasma glucose, insulin, lipid profile, or NO concentrations. However, plasma L-arginine concentrations and L-arginine/ADMA ratios significantly increased in the WM groups compared to the control (*p* = 0.009) after adjusting for age, BMI, race, and gender in the statistical model. **Conclusion**: Overall, BP was not significantly different after two different doses of watermelon compared to control; however, improvements in NO synthesis pathway precursors (L-arginine, ADMA) suggest potential for dietary modulation to support endothelial function and BP regulation.

## 1. Introduction

Hypertension is a major risk factor for cardiovascular diseases (CVDs), including stroke, heart failure, and coronary artery disease. Early intervention is critical since hypertension progresses through several stages, with elevated blood pressure (formally referred to as pre-hypertension) as one of the initial stages characterized by blood pressure (BP) levels higher than the normal range (i.e., <120/80 mm Hg) but below the clinical threshold for hypertension [1]. If left untreated, elevated BP may progress to hypertension, necessitating medication. Several studies have demonstrated that lifestyle changes, including dietary interventions, can be used to manage high BP [2].

Watermelon (*Citrullus lanatus*) is the principal dietary source of L-citrulline, which is a potential biomarker of watermelon consumption [3]. Both L-citrulline and L-arginine have been shown to improve endothelium-dependent, nitric oxide (NO)-mediated vasodilation [4,5,6,7,8]. However, they are metabolized differently in mammals. For example, L-arginine is a semi-essential amino acid which is obtained from dietary sources and a small amount (5–15%) is produced in the body from L-citrulline [9,10]. It also undergoes extensive pre-systemic and systemic elimination from the body by arginases in the gut and liver as well as by gut bacteria [11,12]. In contrast, L-citrulline does not undergo pre-systemic elimination but is metabolized into L-arginine via the sequential actions of arginosuccinate synthase (ASS) and arginosuccinate lyase (ASL) [13]. The L-arginine serves as a substrate for endothelial nitric oxide synthase (eNOS), an enzyme that catalyzes the production of NO. The NO produced by endothelial cells relaxes vascular smooth muscles, resulting in vasodilation, and helps to regulate vascular tone and blood pressure [14]. Human and animal studies have shown that ASL deficiency can cause impairment in endothelial-dependent NO production, which plays a crucial role in the pathogenesis of hypertension [15,16]. These NO-mediated vasodilatory effects have led to growing interest in exploring the potential role of watermelon as a dietary source in managing hypertension and associated cardiovascular risks. Previous clinical studies have demonstrated BP-lowering effects of watermelon supplementation enriched with L-citrulline and L-arginine (typically 4–6 g in a 2:1 ratio) in pre-hypertensive and hypertensive individuals [17,18,19,20]. These antihypertensive effects are primarily attributed to the vasodilatory properties of L-citrulline, which serves as a precursor for L-arginine and enhances NO production, thereby promoting vascular relaxation. In addition to L-citrulline, watermelon contains vitamins A and C, which may contribute to cardiovascular benefits by mitigating oxidative stress and inflammation. Several clinical studies have also reported increased plasma L-citrulline concentrations following watermelon consumption, supporting its potential role in modulating vascular function [21,22,23,24]. However, the concentrations of L-citrulline and L-arginine in a typical serving of fresh watermelon are very low compared to the amounts provided by dietary supplements. Furthermore, there is limited research evaluating the effect of watermelon flesh consumption on BP. Therefore, the primary objective of this study was to investigate whether daily consumption of watermelon flesh, administered at two different doses (1 cup or 2 cups) over a four-week period, could modulate 24 h ambulatory BP in individuals with elevated BP. Secondary objectives included assessing clinical metabolic markers and dietary analytes from watermelon and related biomarkers influencing vasorelaxation.

## 2. Materials and Methods

### 2.1. Study Design and Clinical Trial

**Figure 1:**
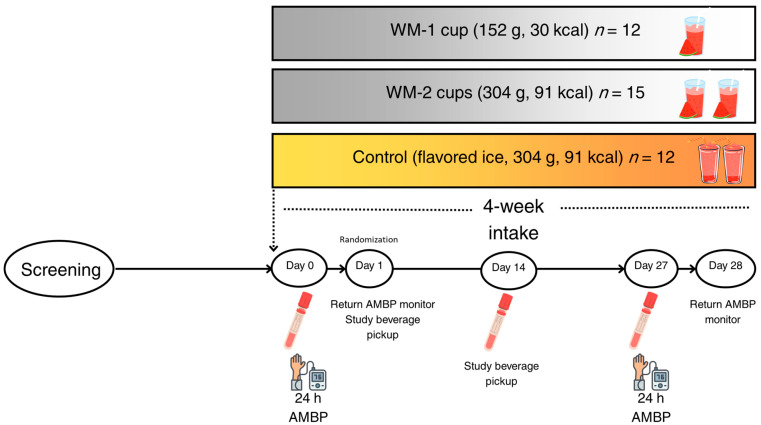
Study design and schema. WM—watermelon flesh; AMBP—ambulatory blood pressure.

This pilot clinical trial was conducted at the Illinois Institute of Technology (Illinois Tech) from April to November 2023. The study was approved by Illinois Tech’s IRB (IRB-2023-03) and registered at ClinicalTrials.gov (NCT05892328). The study was a randomized, single-blinded, placebo-controlled, 4-week parallel clinical trial. Participants were randomly assigned to one of three groups as outlined in the intervention section: a control group, a low-dose watermelon group, and a high-dose watermelon group (Figure 1). The eligibility criteria included non-smoking adults aged 25–60 years with systolic BP ≥ 120–139 mm Hg or a diastolic BP ≥ 80–89 mm Hg and fasting glucose < 120 mg/dL with no chronic diseases, and not taking any medication or dietary supplements affecting the study results (e.g., cholesterol-lowering drugs, L-citrulline, L-arginine, or garlic).

Participants were recruited from the Chicago area via phone, online surveys, or by contacting individuals who had previously participated in studies at the Center for Nutrition Research (CNR). All potential participants were informed about the study procedures through an IRB-approved informed consent form with verbal explanation. Participants who signed the IRB-approved informed consent form proceeded with initial study procedures, which included anthropometric measurements (i.e., weight, height, and waist circumference), body composition analysis, body temperature assessment, fasting glucose measurement via finger prick, and blood pressure measurements.

Participants were scheduled for study visits according to their availability, and they were provided with study instructions, including guidance on how to fill out a three-day diet record before their study visits. Throughout the study period, participants were asked to avoid polyphenol-rich foods and L-citrulline-containing foods (e.g., melons, green leafy vegetables, including arugula and spinach, and pumpkin seeds).

The study participants came to the CNR five times, which included 3 study visits to collect blood and measure BP and 2 visits for study material pickup/return (Figure 1). On day 0, participants arrived at the clinic in a fasted condition, underwent anthropometric measurements, and their food records were reviewed. After baseline blood collection, participants were fitted with an ambulatory blood pressure (AMBP) monitor on their non-dominant arm to record blood pressure over a 24 h period. Day 1 was a follow-up visit, in which the AMBP monitor was returned to the study personnel and data were downloaded to a local computer. Participants were then randomized to one of three study beverages and given enough beverages for the next 2 weeks to consume every morning between 8–10 a.m. At mid-study (day 14), beverage intake was reviewed, blood samples were collected to assess for L-citrulline and L-arginine, and participants picked up study beverages for the remainder of the study duration. Days 27 and 28 involved the same protocol as days 0 and 1, respectively.

### 2.2. Study Interventions

Seedless watermelons were purchased from Costco (Chicago, IL, USA). Watermelons were washed, cut, and the flesh was separated in the CNR metabolic kitchen following good manufacturing practices. The cut watermelons were packed into Ziploc bags and stored in a −20 °C freezer until use. The frozen watermelons were thawed and blended to a beverage using a Ninja Blender (Model CO351B, Shark Ninja Operating LLC, Needham, MA, USA). Participants were randomized to consume daily for 4 weeks one of three beverages: control (0 g WM), WM-1 cup (152 g), or WM-2 cups (304 g). The control beverage was prepared using watermelon-flavored Italian ice, which matched the calorie content of the WM 2-cups. The nutrient composition of interventions was analyzed using the Food Processor Nutrition and Fitness Software by Esha Research version 11.11.32 (Table 1).

### 2.3. Ambulatory Blood Pressure Assessment

Ambulatory BP was measured over 24 h on day 0 and day 28 using an AMBP monitor (Contec Medical Systems Co. Ltd., Model 50, Qinhuangdao, Hebei Province, China). Participants wore the monitor on their non-dominant arm, and BP readings were automatically recorded every hour over a 24 h period. The daytime BP period was defined between 10:00 a.m. and 8:00 p.m., and the nighttime period was defined between 12:00 a.m. and 6:00 a.m. [25]. The fixed-time approach was chosen for capturing diurnal variation in BP because it is a reasonable alternative when self-report data are limited [26]. Three study investigators reviewed the data independently, identifying and removing outlier values, including invalid readings due to loose cuffs. Participants were classified as having a complete AMBP recording if they fulfilled the criteria established by the International Database of Ambulatory Blood Pressure Monitoring and Cardiovascular Outcomes (IDACO), defined as obtaining at least 10 systolic and diastolic BP readings during the daytime (10:00 a.m. to 8:00 p.m.) and at least 5 readings during the nighttime (midnight to 6:00 a.m.) [27]. All participants were categorized as non-dipper as none exhibited a decrease in systolic and diastolic BP exceeding 10% [28].

### 2.4. Analysis of Glucose, Insulin, and Lipid Profile in Plasma

Blood samples were collected in EDTA tubes and immediately placed on ice. Plasma was separated by centrifuging for 15 min at 453× *g*, aliquoting 100–450 µL into individual vials, and stored at −80 °C until further analysis. Plasma was analyzed for glucose, insulin, triglycerides (TG), total cholesterol (TC), and high-density lipoprotein cholesterol (HDL-C) using an RX Daytona automated clinical analyzer (Randox Laboratories, Crumlin, UK) with appropriate internal and external control samples. Low-density lipoprotein cholesterol (LDL-C) was calculated using the Friedewald equation [29].

### 2.5. Quantification of Select Amino Acids in Watermelon Flesh and Plasma

#### Chemicals and Materials

HPLC grade acetonitrile, methanol, isopropanol, formic acid, ammonium formate, formic acid, L-citrulline, L-arginine, and 0.45 µm polytetrafluoroethylene (PTFE) filters were purchased from Fisher Scientific (Pittsburgh, PA, USA). Blank human plasma (pooled, gender unspecified, 0.2 µm filtered, charcoal-stripped) was purchased from BIOIVT Elevating Science (Hicksville, NY, USA). Ultrapure water was produced using a Millipore Direct-Q 3 Water Purification System (Burlington, MA, USA).

Extraction of L-Citrulline and L-Arginine from the Watermelon Flesh: Compounds in watermelon flesh were extracted and analyzed using a previously published method from our lab [21]. Watermelon flesh (5 g) was extracted three times with 80% methanol. The samples were vortexed for 30 s, followed by sonication for 30 min in chilled water. The extracts were centrifuged at 8228× *g* for 10 min at 7 °C, the supernatants were collected and combined after three extractions, and the final volume was made up to 35 mL. Out of the collected extract, 50 µL was dried under nitrogen gas and reconstituted in 1 mL of 50% methanol for the ultra-high-performance liquid chromatography-triple quadrupole mass spectrometry analysis (UHPLC-QQQ-MS/MS).

Extraction of L-Citrulline, L-Arginine, and Asymmetric Dimethylarginine (ADMA) from Plasma: Plasma (200 μL) was thawed and extracted with 600 μL of acidified isopropanol (0.1% formic acid). The samples were vortexed for 30 s and centrifuged at 18,514× *g* for 10 min. The supernatants were filtered through 0.45 μm PTFE syringe filters before UHPLC-QQQ-MS/MS analysis [21].

UHPLC-QQQ-MS/MS Analysis: The extracted watermelon flesh and plasma samples were analyzed on an Agilent 1290 Infinity UHPLC system coupled with a 6460 Triple Quadrupole Tandem Mass Spectrometer (UHPLC-QQQ-MS/MS) equipped with positive electrospray ionization (ESI) and operated in multiple-reaction monitoring (MRM) mode (Agilent Technologies, Santa Clara, CA, USA). Mass spectrometric parameters included a drying gas flow rate of 9 L/min at 200 °C and a sheath gas flow rate of 11 L/min at 300 °C. The chromatographic separation was performed using an Infinity Lab Poroshell 120 HILIC-Z column (2.1 × 100 mm, 2.7 μm, Agilent Technologies, Newport, DE, USA) equipped with a matching guard column (2.1 × 5 mm, 2.7 μm, Agilent Technologies, Newport, DE, USA) maintained at a constant temperature of 25 °C with a flow rate of 0.3 mL/min and an injection volume of 1 μL. Mobile phases were prepared using a stock solution of 200 mM of ammonium formate in water (pH adjusted to 3 with formic acid). Mobile phase A consisted of 20 mM ammonium formate in water (pH 3), and B consisted of 20 mM ammonium formate in 90% acetonitrile (pH 3). The mobile phase gradient was programmed from 100–70% of mobile phase B over 11.5 min and back to 100% of mobile phase at 12 min. A 5 min post-run period was included in the gradient to allow the column to re-equilibrate to the initial mobile phase conditions. The data were analyzed using MassHunter quantitative analysis software (version B.07.00, Agilent Technologies, Santa Clara, CA, USA). L-citrulline and L-arginine concentrations were quantified using calibration curves generated from their respective reference standards in blank plasma. ADMA was quantified using the calibration curve generated for L-arginine.

### 2.6. Plasma NO Concentrations

Plasma NO concentrations were quantified using the commercial colorimetric assay kit following the manufacturer’s recommended procedures (Elabscience Metabolism Assay Kit, Catalog# E-BC-K035-M, Houston, TX). Given the short half-life and instability of NO in physiological systems, the assay measures its stable oxidation product nitrite (NO_2_^−^) as an indicator of NO production. NO was measured using a microplate reader (GloMax Discover, Promega, Madison, WI, USA) at 540 nm absorbance. Plasma NO concentrations were calculated based on a standard curve generated from known nitrite concentrations using a linear regression equation.

### 2.7. Statistical Analysis

Data from clinical biomarkers and chemistry analysis were statistically analyzed using IBM SPSS Statistics (Version 29.0.0). All data were tested for normality before statistical analysis using histograms, Q-Q Plots, and normality tests (Kolmogorov–Smirnov and Shapiro–Wilk). Univariate analyses of variance (UNIANOVA) were conducted to examine the effects of study interventions after 4 weeks on plasma glucose and insulin markers, lipid profile, NO, L-arginine, L-citrulline, ADMA, and L-arginine/ADMA ratio. Baseline (week 0) values were included as covariates in each model (analysis of covariance, ANCOVA). Additional analyses were conducted by including demographic covariates such as age, BMI, race, and gender in the statistical model. A repeated measures general linear model (RM-GLM) was used to assess within-subject changes over time and to examine time × intervention interactions for all the clinical biomarkers and chemistry data. Baseline characteristics across study intervention groups were compared using one-way analysis of variance (ANOVA). No formal power calculation was performed a priori to determine sample size, as this was a pilot exploratory study. Our goal was to have at least 12 individuals per group complete the study with evaluable data. All statistical tests were two-tailed, and significance was determined at *p* < 0.05.

## 3. Results

### 3.1. Participant Enrollment, Demographics, and Baseline Characteristics

**Figure 2:**
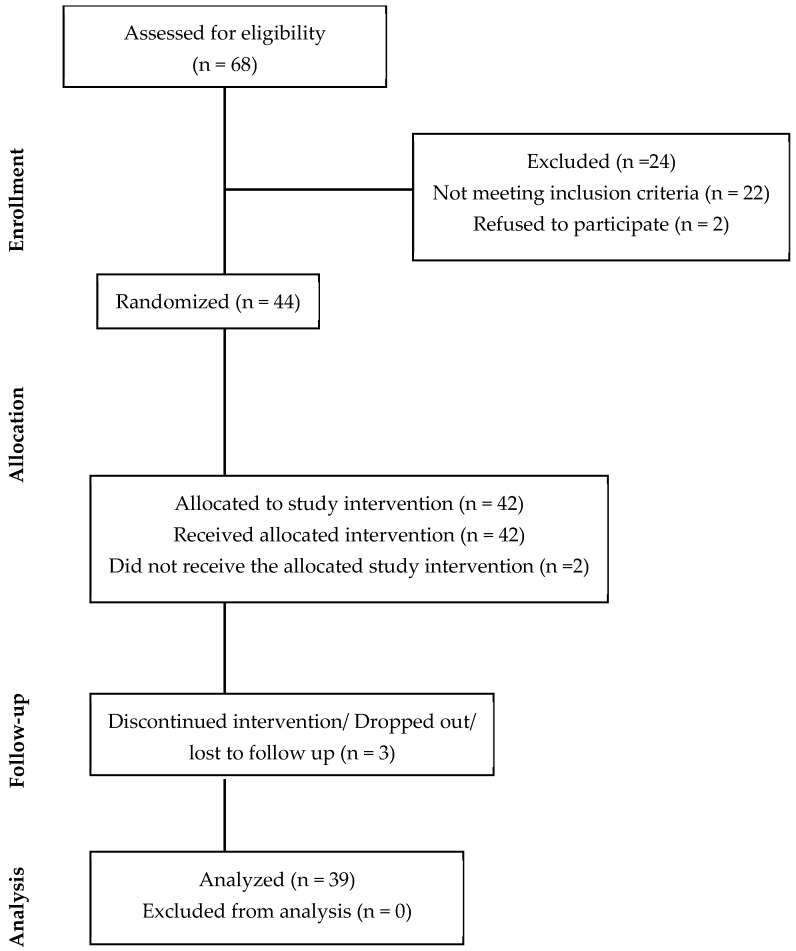
Consolidated Standards of Reporting Trials (CONSORT) diagrams for study recruitment.

Around 100 individuals filled out the online screening questionnaire, out of which 68 qualified for a pre-screening visit at CNR. A total of 44 participants were randomized and enrolled in the study. Five individuals dropped out of the study due to a lack of follow-up, resulting in 39 participants who completed the study (CONSORT diagram, Figure 2).

Baseline demographic characteristics were comparable across intervention groups. The average age (mean ± SD) was 35 ± 12 y, 41 ± 12 y, and 43 ± 12 y and BMI was 28.4 ± 1.5 kg/m^2^, 31.9 ± 1.3 kg/m^2^, and 30.6 ± 2.1 kg/m^2^ in the control, WM-1 cup, and WM-2 cups groups, respectively, and not statistically different between groups (*p* > 0.05). Likewise, BP and fasting blood glucose concentrations were not statistically different among intervention groups (*p* > 0.05) (Table 2).

### 3.2. L-Citrulline and L-Arginine Content in Study Beverages

The concentration of L-citrulline and L-arginine in watermelon flesh was 4.85 ± 0.3 mg/g and 0.54 ± 0.0 mg/g, respectively. Accordingly, the concentration of L-citrulline and L-arginine in WM-1 cup beverage (low-dose, 152 g) was 737.8 ± 34.8 mg and 82.0 ± 5.8 mg, respectively. The WM-2 cups beverage (high-dose, 304 g) contained 1475.6 ± 69.7 mg of L-citrulline and 164.0 ± 11.6 mg of L-arginine. L-citrulline and L-arginine were not detectable in the control beverage (Table 3).

### 3.3. Assessment of Ambulatory Blood Pressure

Ambulatory BP was recorded at baseline (week 0) and post-intervention (week 4) for 24 h. Analyses of 24 h, daytime, nighttime, and morning surge are reported in Table 4. At baseline, the average 24 h systolic/diastolic BP was similar across groups. After 4 weeks, systolic BP changes were −1.8 ± 2.9 mm Hg (control), −1.1 ± 2.8 mm Hg (WM-1 cup), and −3.2 ± 1.9 mm Hg (WM-2 cups); diastolic BP changes were −2.8 ± 2.0 mm Hg (control), −0.8 ± 3.7 mm Hg (WM-1 cup), and −0.8 ± 1.6 mm Hg (WM-2 cups). No significant differences were observed among groups for 24 h, daytime, nighttime, or morning surge BP measures (*p* > 0.05).

### 3.4. Plasma Analyses

#### 3.4.1. Glucose, Insulin, and Lipid Profile

**Figure 3:**
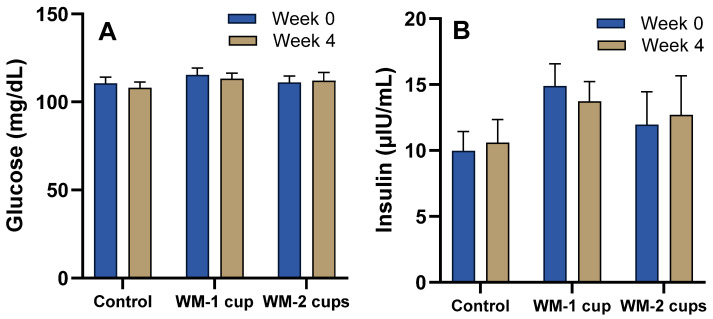
Fasting plasma glucose (**A**) and insulin (**B**) concentrations after 4 weeks of daily consumption of either low-dose watermelon beverage (WM-1 cup; 152 g, 30 kcal; n = 15), high-dose watermelon beverage (WM-2 cups; 304 g, 91 kcal; n = 12), or a calorie-matched control beverage (watermelon-flavored Italian ice; 304 g, 91 kcal; n = 12) in adults with elevated blood pressure. Values are presented as mean ± standard error of the mean (SEM). No statistically significant intervention effect (*p* > 0.05) was observed.

Fasting plasma glucose concentrations at baseline (week 0) were 110.6 ± 3.5 mg/dL, 115.3 ± 4.1 mg/dL, and 111.1 ± 3.4 mg/dL in control, WM-1 cup, and WM-2 cups groups, respectively, and did not change significantly after any of the interventions (*p* > 0.05) (Figure 3A). Similarly, fasting plasma insulin concentrations at baseline (week 0) were 10.0 ± 1.5 μIU/mL, 14.9 ± 1.7 μIU/mL, and 12.0 ± 2.4 μIU/mL in the control, WM-1 cup, and WM-2 cups groups, respectively, and did not differ among groups after 4 weeks’ intervention (*p* > 0.05) (Figure 3B).

**Figure 4:**
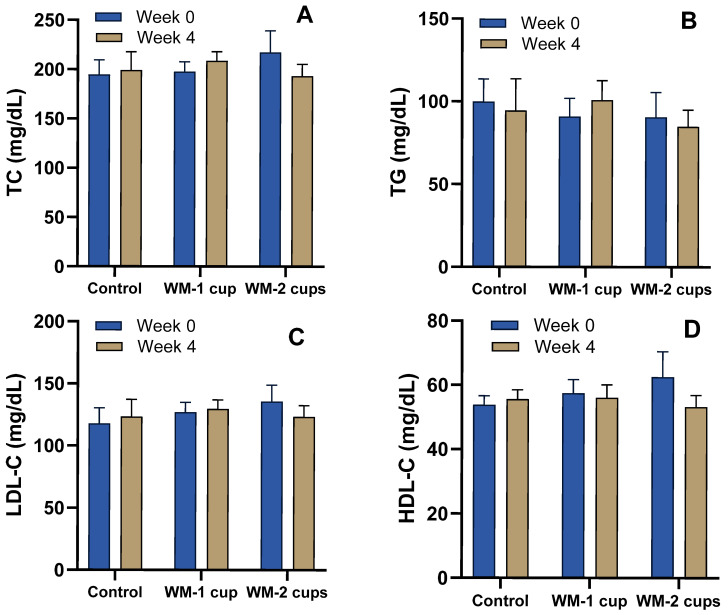
Fasting plasma lipid profile after 4 weeks of daily intake of low-dose watermelon beverage (WM-1 cup; n = 15), high-dose watermelon beverage (WM-2 cups; n = 12), or a calorie-matched control beverage (n = 12) in adults with elevated blood pressure. Panels show total cholesterol (TC) (**A**), triglycerides (TG) (**B**), low-density lipoprotein cholesterol (LDL-C) (**C**), and high-density lipoprotein cholesterol (HDL-C) (**D**). Data are presented as means ± SEM. No statistically significant intervention effect (*p* > 0.05) was observed.

TC decreased in the WM-2 cups group (−21.6 ± 16.9 mg/dL) but increased in the WM-1 cup and control groups. TG concentrations showed small reductions in WM groups, most notably in WM-1 cup (−7.3 ± 7.0 mg/dL). Both LDL-C (−10.0 ± 10.2 mg/dL) and HDL-C (−7.9 ± 7.4 mg/dL) decreased in the WM-2 cups group and increased in the control group (Figure 4). Despite apparent changes, no significant differences were observed in plasma lipid profiles (TC, TG, LDL-C, HDL-C) among groups after 4 weeks (*p* > 0.05).

#### 3.4.2. Watermelon-Specific Amino Acids, ADMA, and L-Arginine/ADMA Ratio

**Figure 5:**
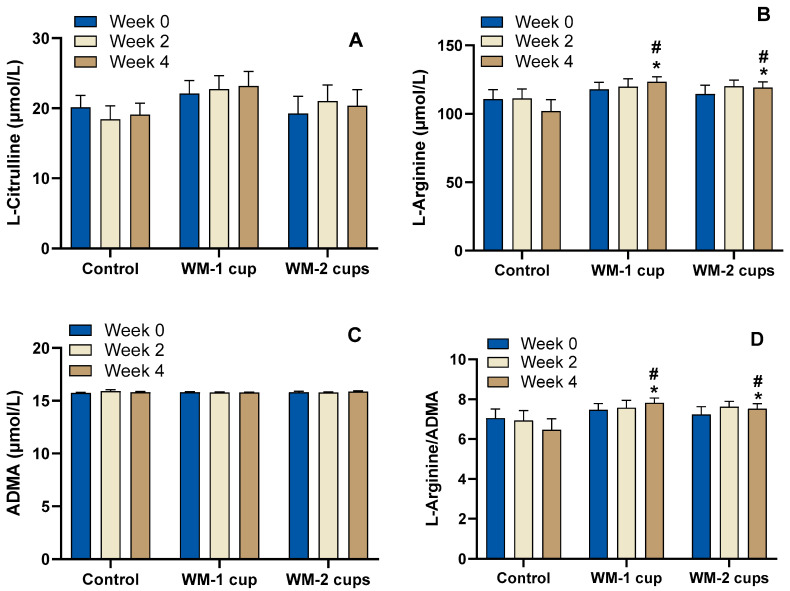
Fasting plasma concentrations of L-citrulline (**A**), L-arginine (**B**), asymmetric dimethylarginine (ADMA) (**C**), and L-arginine/ADMA ratio (**D**) after 4 weeks of daily consumption of low-dose watermelon beverage (WM-1 cup; n = 15), high-dose watermelon beverage (WM-2 cups; n = 12), or a calorie-matched control beverage (n = 12) in adults with elevated blood pressure. Data are presented as mean ± SEM. No statistically significant intervention effect (*p* > 0.05) was observed for L-citrulline. L-arginine showed a marginal intervention effect (*p* = 0.081), which became significant after adjusting for covariates (intervention: *p* = 0.009; BMI: *p* = 0.009) using ANCOVA. No statistically significant differences (*p* > 0.05) were observed for plasma ADMA concentrations among the study intervention groups. The L-arginine/ADMA ratio showed a marginal intervention effect (*p* = 0.083), which became significant after covariate adjustment (intervention: *p* = 0.009; BMI: *p* = 0.008) using ANCOVA. * indicates marginal significance; # indicates significance after adjusting for covariates (age, BMI, gender, and race).

The concentrations of L-citrulline and L-arginine were determined in fasting plasma at baseline (week 0), mid-study (week 2), and end of the study (week 4) (Figure 5).

L-citrulline: Fasting plasma concentrations of L-citrulline remained relatively stable throughout the study period, with modest increases observed after WM beverage intake. For example, there was ~5% increase in plasma L-citrulline (week 0, 22.1 ± 2.0 µmol/L to week 4, 23.2 ± 2.0 µmol/L) in participants who consumed 1 cup of WM beverage, and ~ 6% increase (week 0, 19.3 ± 2.4 µmol/L to week 4, 20.4 ± 2.3 µmol/L) in participants who consumed 2 cups of WM beverage for 4 weeks. In contrast, the control group showed a decrease of ~15% in L-citrulline concentrations over 4 weeks (Figure 5A). Differences at week 4 were not statistically significant among the different study intervention groups (*p* > 0.05) in the ANCOVA analysis, even after adjusting for covariates (race, *p* = 0.003). A main effect of time (*p* = 0.007) was observed in the RM-ANOVA, with no significant effect of intervention or intervention by time interaction (*p* > 0.05).

L-arginine: L-arginine concentrations increased modestly (~4.5%) from week 0 to week 4 in participants who consumed WM-1 cup and WM-2 cups of beverages, whereas L-arginine concentrations reduced by ~8% in the control beverage group (Figure 5B). L-arginine concentrations were marginally different among intervention groups at week 4 (*p* = 0.081) and became significant (*p* = 0.009) after adjusting for covariates (age, BMI, gender, and race), of which BMI was significant (*p* = 0.009).

ADMA: Baseline plasma ADMA levels were similar across groups (~15.7–15.8 μmol/L) and remained unchanged throughout the study (Figure 5C) (*p* > 0.05). In the between-subjects analysis only race was significant in the model (*p* = 0.028).

The L-arginine/ADMA ratio increased slightly in both WM intervention groups (WM-1 cup: from 7.5 ± 0.3 to 7.8 ± 0.2; WM-2 cups: from 7.2 ± 0.4 to 7.5 ± 0.3), while it decreased in the control group after 4 weeks (Figure 5D). In the baseline-adjusted ANCOVA, intervention group differences at week 4 were marginally significant (*p* = 0.083), and reached significance (*p* = 0.009) after including covariates (BMI, *p* = 0.008). These data are consistent with the results of L-arginine.

#### 3.4.3. Nitric Oxide (NO)

**Figure 6:**
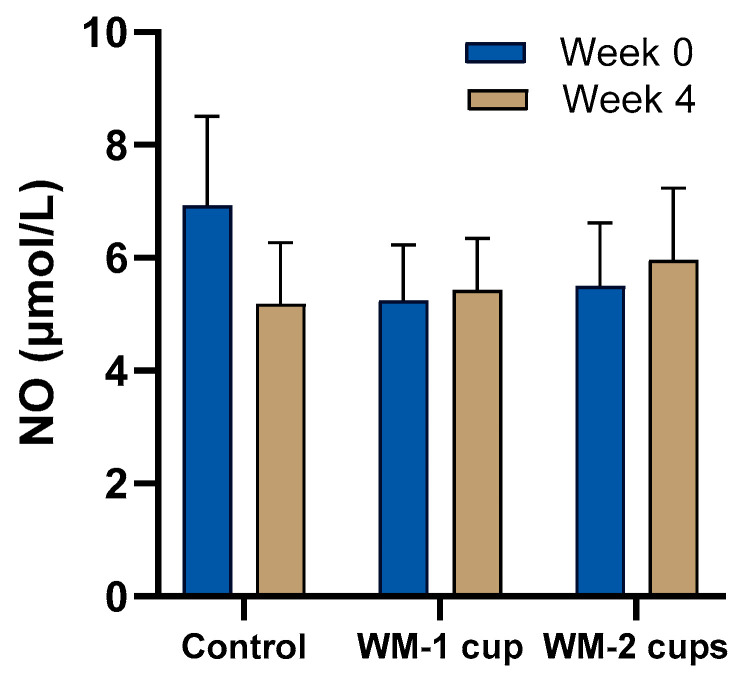
Fasting plasma nitric oxide (NO) concentrations after 4 weeks of daily intake of low-dose watermelon beverage (WM-1 cup; n = 15), high-dose watermelon beverage (WM-2 cups; n = 12), or a calorie-matched control beverage (n = 12) in adults with elevated blood pressure. Data are presented as means ± SEM. No statistically significant intervention effect (*p* > 0.05) was observed.

NO concentrations slightly increased from week 0 (baseline) to week 4 in both the WM groups, i.e., from 5.2 ± 0.1 µmol/L to 5.4 ± 0.9 µmol/L in the WM-1 cup group and from 5.6 ± 1.1 µmol to 5.9 ± 1.3 µmol/L in the WM-2 cups group. NO concentrations decreased from 6.9 ± 1.5 µmol/L to 5.2 ± 1.0 µmol/L in the control group after 4 weeks (Figure 6). Despite these trends, no statistically significant effects (*p* > 0.05) were observed for intervention groups on NO levels after 4 weeks, even after adjusting for covariates.

## 4. Discussion

This randomized placebo-controlled pilot clinical trial investigated the impact of two doses of WM flesh beverages (1 cup and 2 cups daily) on 24 h ambulatory BP in adults with elevated BP over a 4-week period. In addition to ambulatory BP, the study also assessed circulating concentrations of glucose, insulin, blood lipids (TC, TG, LDL-C, HDL-C,), NO, L-arginine, L-citrulline, and ADMA to explore potential mechanisms of vascular modulation.

Watermelon is a natural source of L-citrulline, which ranges from 0.9 to 4.3 mg/kg of fresh fruit [21,30]. L-citrulline is a precursor of L-arginine, a substrate for eNOS that catalyzes the production of NO, which is responsible for maintaining vascular tone and BP. Our results showed a non-significant decrease in both systolic and diastolic 24 h ambulatory BP after 4 weeks of regular intake of WM. This is contrary to the previous literature suggesting the beneficial effect of watermelon supplementation enriched with L-citrulline/L-arginine and WM-derived products on BP [17,18,19,20,31,32]. A recent meta-analysis of randomized clinical trials reported that watermelon supplementation resulted in a significant reduction in systolic BP (−10.55 mm Hg; 95% CI: −15.30 to −5.80) and diastolic BP (−5.22 mm Hg; 95% CI: −9.82 to −0.62) [33].

The major difference between the current study and the previous literature on watermelon with significant changes in BP is the dose and form of watermelon used. Many prior studies have used concentrated watermelon extracts or freeze-dried powders with substantially higher concentrations of L-citrulline and L-arginine compared to those found in watermelon flesh. For example, a pilot study conducted in pre-hypertensive individuals showed that watermelon supplementation in the form of freeze-dried extract (L-citrulline/L-arginine: 1.35 g/0.65 g two times per day) or sugar-matched placebo as control for 6 weeks could help reduce aortic systolic BP [19]. However, compared to our study, this research group provided 2.7 g of L-citrulline per day, which was ~2–3.5 times higher than our study doses (WM-1 cup containing 0.74 g L-citrulline and WM-2 cups 1.5 g of L-citrulline). The same research group reported decreased aortic systolic and diastolic BP in obese hypertensive or pre-hypertensive adults and postmenopausal women after 6 weeks’ consumption of freeze-dried watermelon extract (6 g/day with L-citrulline/arginine ratio of ~2:1) [17,18,20]. Lum et al. (2019) reported a significant reduction in systolic BP after 4 weeks’ supplementation of 2 cups of watermelon flesh in overweight or obese individuals (n = 33) using a cross-over study design [31]. Even though our study was conducted using watermelon flesh, we did not observe any significant changes in BP which could be due to variability in study participants, study design, and small sample size.

We did not observe any significant changes in cardiometabolic risk markers (glucose, insulin, TC, LDL-C, HDL-C, and TG) after 4-week intake of WM beverages at two different dosages (1 cup and 2 cups) compared to control, which aligns with findings from other chronic feeding studies on watermelon and cardiometabolic risk markers [31,34,35]. A meta-analysis of randomized controlled trials on watermelon consumption reported that longer-term watermelon consumption may improve vascular function, without any changes in postprandial or fasting cardiometabolic risk markers such as glucose and TC [35]. In a randomized, double-blind, placebo-controlled cross-over trial involving healthy postmenopausal women (n = 21), supplementation with two daily servings of 360 mL of watermelon juice for 4 weeks did not show any effect on vascular function or glucose homeostasis [34]. Lum et al., 2019 observed no significant changes in postprandial glucose and insulin responses after 4 weeks’ consumption of 2 cups of fresh WM in overweight and obese adults [31]. Similarly, Daughtry et al. reported no changes in glucose, insulin, and lipid profile after intake of blended watermelon juice (1 cup of blended watermelon flesh and rind, 240 g) or an isocaloric sugar-sweetened beverage in an 8-week randomized, cross-over clinical trial in overweight or obese children [36]. Data from this study along with others indicate that incorporating up to 2 cups of WM in the daily diet for 4 weeks had no adverse effects on glucose homeostasis or lipid profiles.

Watermelons are distinctly rich in two non-proteinaceous amino acids L-citrulline and L-arginine, along with carotenoids, (poly)phenols, vitamin C, sugars, and minerals [21,30,37,38,39,40]. In this study, participants consumed WM flesh beverages daily for 4 weeks at two different doses. The low-dose beverage (WM-1 cup/day) delivered 738 mg of L-citrulline and 82 mg of L-arginine, while the high-dose (WM-2 cups/day) provided 1476 mg of L-citrulline and 164 mg of L-arginine. The control beverage contained neither amino acid. The concentrations of L-citrulline and L-arginine in WM flesh are comparable to previously reported values in the literature [41].

L-citrulline is metabolized in the body to form L-arginine, which serves as a key precursor for the synthesis of NO, the signaling molecule involved in numerous biological processes, including vasodilation and BP regulation. Through the arginine–citrulline–NO cycle, L-citrulline contributes to sustained L-arginine availability and NO production [42]. ADMA, on the other hand, is an endogenous competitive inhibitor of NO synthase and is a risk factor for endothelial dysfunction [43].

Both L-citrulline and L-arginine from watermelon are bioavailable and achieve maximum concentrations in blood between 1–2 h [21]. In a previous cross-over clinical trial, fasting plasma L-arginine and ornithine concentrations increased in healthy adults following daily consumption of watermelon juice containing either 1 g (low-dose) or 2 g of L-citrulline (high-dose) for 1 week and 3 weeks compared to control juice (0 g L-citrulline) [44]. Likewise, Shanely et al. (2020) reported about 8% increase in fasting L-arginine concentration after consuming 1.88 g L-citrulline and 0.40 g L-arginine per day in the form of WM puree for 6 weeks in overweight/obese postmenopausal women [45]. Consistent with previous findings, we observed an increase in fasting plasma concentrations of L-citrulline, L-arginine, and L-arginine/ADMA ratio after 4 weeks of daily WM beverage intake. These results reinforce the potential of watermelon-derived L-citrulline to support NO-related physiological functions through enhanced availability of L-arginine.

Our results also indicate that individual characteristics such as BMI, race, and age may contribute to variability in L-citrulline, L-arginine, and ADMA responses which is supported by previously published research [10,46,47,48,49,50]. These demographic variables should be considered in future studies to improve the precision of dietary interventions.

We observed a modest, non-significant increase in NO in the WM intervention groups compared to the control. Watermelon is abundant in L-citrulline, which serves as a substrate for L-arginine synthesis in endothelial cells, leading to increased production of NO. In a cross-over clinical trial in healthy adult males (n = 8), supplementation with 300 mL of watermelon juice concentrate (~3.4 g L-citrulline) for 2 weeks increased fasting plasma concentrations of nitrite [22]. It is to be noted that the L-citrulline content in the watermelon juice concentrate used in that study was more than twice the amount present in the 2 cups of watermelon flesh provided in the current study.

## 5. Conclusions

This was the first clinical trial to assess the effects of daily intake of two different doses of watermelon flesh for 4 weeks on 24 h ambulatory BP, along with glucose, insulin, lipid profile, and select vascular function biomarkers in adults with elevated blood pressure. Our findings suggest that watermelon intake may favorably influence vascular health, as indicated by significant improvements in L-arginine availability and the L-arginine/ADMA ratio. However, observation of significant effects may have been limited by the relatively short intervention period, small sample size, and relatively low L-citrulline dosage achievable from whole fruit compared to higher doses used in enriched interventions published previously [17,18,19]. The outcomes could also vary depending on individual physiological responses (demographic factors) to watermelon intake and the form of L-citrulline/L-arginine provided (supplements or extracts compared to whole fruit). It is possible that a higher intake might be necessary to impact blood pressure and associated pathways underlying vascular health.

Our research contributes to understanding how modest dietary interventions influence cardiovascular-related endpoints, especially in at-risk populations. To strengthen and expand on these findings, future studies should consider a larger sample size, controlling for demographic variables, with a longer intervention period of 8–12 weeks, comparison of different forms of interventions (juice, extracts, supplements vs. whole fruit), and inclusion of broader range of vascular biomarkers to understand the mechanism involved.

## Figures and Tables

**Table 1:** Nutritional composition of study beverages ^1^.

Nutrients	Control (304 g)	WM-1 Cup (152 g)	WM-2 Cups (304 g)
Energy (kcal)	~91	~30	~91
Carbohydrate (g)	18.85	7.70	23
Total sugar (g)	18.85	6.32	18.85
Total fat (g)	0	2	4
Added sugar (g)	18.85	0	0
Saturated fat (g)	0	0.02	0.5
Protein (g)	0	1.85	2
Total fiber (g)	0	0.41	1
Soluble fiber (g)	0	0.10	0.30

^1^ Analyzed with Esha software (version 11.11.32).

**Table 2:** Baseline characteristics of study participants.

Characteristics ^1^	Control (n = 12)	WM-1 Cup (n = 15)	WM-2 Cups (n = 12)
Age, years	35 ± 12	41 ± 15	43 ± 14
Sex, F/M, n	15/8	7/8	9/14
AS:HIS:CAU:AA	6:0:1:5	2:1:5:7	0:2:5:5
BMI (kg/m^2^)	28.4 ± 1.5	31.9 ± 1.3	30.6 ± 2.1
Systolic blood pressure, mm Hg	127.1 ± 2.2	127.1 ± 2.8	123.3 ± 2.2
Diastolic blood pressure, mm Hg	85.1 ± 1.8	80.7 ± 1.9	79.5 ± 1.6
Fasting blood glucose—Capillary mg/dL	103.3 ± 3.2	109.9 ± 2.9	103.6 ± 2.3

^1^ Data are mean ± SEM except age (mean ± SD); AA, African American; AS, Asian; CAU, Caucasian; HIS, Hispanic. No statistical differences were observed in baseline characteristics such as age, BMI, systolic blood pressure, diastolic blood pressure, and fasting blood glucose (*p* > 0.05).

**Table 3:** L-citrulline and L-arginine concentrations in study beverages.

Samples	L-Citrulline	L-Arginine
WM flesh (mg/g)	4.85 ± 0.3	0.54 ± 0.0
WM-1 cup beverage (mg/152 g)	737.8 ± 34.8	82.0 ± 5.8
WM-2 cups beverage (mg/304 g)	1475.6 ± 69.7	164.0 ± 11.6
Control beverage (mg/304 g)	ND	ND

Values are means ± SD; n = 3; ND—not detected.

**Table 4:** Blood pressure data before and after 4 weeks of study interventions.

AMBP Data (Average—mm Hg)	Control (n = 12)	Control (n = 12)	WM-1 Cup (n = 15)	WM-1 Cup (n = 15)	WM-2 Cups (n = 12)	WM-2 Cups (n = 12)
	Systolic	Diastolic	Systolic	Diastolic	Systolic	Diastolic
**Week 0 (Baseline)**						
24 h BP	131.9 ± 3.2	82.3 ± 2.5	131.2 ± 3.0	79.1 ± 2.3	128.2 ± 3.1	76.8 ± 2.4
Daytime BP	134.3 ± 3.6	86.0 ± 2.9	133.9 ± 2.2	82.5 ± 2.3	130.1 ± 3.1	78.3 ± 2.8
Nighttime BP	127.8 ± 4.4	75.4 ± 3.0	127.3 ± 4.7	74.5 ± 3.2	121.8 ± 4.6	70.6 ± 3.0
Morning surge	−0.4 ± 2.1	2.0 ± 1.7	8.4 ± 2.9	9.3 ± 4.0	2.0 ± 2.5	4.2 ± 2.5
**Week 4**						
24 h BP	130.2 ± 3.9	79.5 ± 3.0	130.1 ± 3.2	78.3 ± 2.1	124.9 ± 3.9	76.1 ± 2.5
Daytime BP	130.0 ± 4.5	81.0 ± 3.2	131.7 ± 3.1	81.7 ± 2.6	127.5 ± 4.1	78.4 ± 2.6
Nighttime BP	123.6 ± 3.1	75.3 ± 2.8	120.0 ± 3.5	70.5 ± 2.8	122.7 ± 5.3	73.3 ± 3.2
Morning surge	3.5 ± 3.5	1.6 ± 2.3	5.8 ± 2.3	7.1 ± 5.5	5.7 ± 2.2	7.0 ± 2.5
**Change (Δ)** **Week 4–Week 0**						
24-h BP	−1.8 ± 2.9	−2.8 ± 2.0	−1.1 ± 2.8	−0.8 ± 3.7	−3.2 ± 1.9	−0.8 ± 1.6
Daytime BP	−4.3 ± 4.5	−5.0 ± 2.8	−2.2 ± 2.9	−0.8 ± 2.6	−3.2 ± 2.0	−0.2 ± 1.6
Nighttime BP	−6.4 ± 3.9	−2.1 ± 2.3	−7.3 ± 4.2	−0.2 ± 4.5	2.3 ± 2.6	3.5 ± 2.0
Morning surge	−2.3 ± 4.3	−1.6 ± 3.1	−0.2 ± 4.5	−3.1 ± 5.5	3.4 ± 3.4	2.4 ± 3.0

WM-1 cup (152 g, 30 kcal, n = 15), WM-2 cups (304 g, 91 kcal, n = 12), and the control beverage (watermelon flavored Italian ice, 304 g, 91 kcal, n = 12). Data are means ± SEM. No statistically significant intervention effect (*p* > 0.05) was observed. AMBP: Ambulatory blood pressure.

## Data Availability

The data presented in this study are available on request from the corresponding author.

## Abbreviations

AA	African American
ADMA	Asymmetric Dimethylarginine
AMBP	Ambulatory Blood Pressure
ANCOVA	Analysis of Covariance
ANOVA	Analysis of Variance
AS	Asian
ASL	Arginosuccinate Lyase
ASS	Arginosuccinate Synthase
BMI	Body Mass Index
BP	Blood Pressure
CAU	Caucasian
CNR	Center for Nutrition Research
CONSORT	Consolidated Standards of Reporting Trials
CVD	Cardiovascular Disease
EDTA	Ethylenediaminetetraacetic Acid
eNOS	Endothelial Nitric Oxide Synthase
ESI	Electrospray Ionization
HDL-C	High-Density Lipoprotein Cholesterol
HIS	Hispanic
HPLC	High-Performance Liquid Chromatography
IDACO	International Database of Ambulatory Blood Pressure Monitoring and Cardiovascular Outcomes
IRB	Institutional Review Board
LDL-C	Low-Density Lipoprotein Cholesterol
MRM	Multiple-Reaction Monitoring
NO	Nitric Oxide
PTFE	Polytetrafluoroethylene
RM-ANOVA	Repeated Measures Analysis of Variance
RM-GLM	Repeated Measures General Linear Model
SD	Standard Deviation
SEM	Standard Error of the Mean
SPSS	Statistical Package for the Social Sciences
TC	Total Cholesterol
TG	Triglycerides
UHPLC-QQQ-MS/MS	Ultra-High-Performance Liquid Chromatography-Triple Quadrupole Tandem Mass Spectrometry
WM	Watermelon
